# Comparative analysis of dapagliflozin and linagliptin in managing type 2 diabetes mellitus and heart failure: A retrospective study

**DOI:** 10.1097/MD.0000000000044709

**Published:** 2025-12-12

**Authors:** Wenxin Zai, Ting Dai, Li Wang, Bo Liu

**Affiliations:** a Department of Cardiovascular, The Sixth Hospital of Wuhan, Affiliated Hospital of Jianghan University, Wuhan, Hubei Province, China; b Department of Geriatrics, The Sixth Hospital of Wuhan, Affiliated Hospital of Jianghan University, Wuhan, Hubei Province, China.

**Keywords:** dapagliflozin, efficacy, heart failure, linagliptin, safety, type 2 diabetes mellitus

## Abstract

This study retrospectively evaluates and compares the clinical effects of dapagliflozin and linagliptin on cardiac function and glycemic control in patients with type 2 diabetes mellitus (T2DM) complicated by heart failure (HF), thereby providing real-world evidence to support clinical decision-making. This retrospective study included 200 patients with both T2DM and HF, who were divided into 2 groups based on their treatment regimens: a control group and a treatment group (100 patients each). All patients received standard anti-HF therapy in combination with metformin. The control group was administered linagliptin (5 mg once daily before breakfast), while the treatment group received dapagliflozin (10 mg once daily) for a duration of 6 months. Relevant clinical data were collected and analyzed for both groups. Baseline characteristics were comparable between the 2 groups (*P* > .05). The total effective rate was significantly higher in the treatment group compared with the control group (97.5% vs 80.0%, *P* < .001), indicating a superior improvement in clinical symptoms with dapagliflozin. Both groups showed significant reductions in fasting blood glucose, 2-hour postprandial blood glucose, and HbA1c compared with baseline values (*P* < .001), with no statistically significant differences between groups (*P* > .05), suggesting that both drugs effectively improve glycemic control. The treatment group demonstrated greater improvements in NT-proBNP, fibroblast growth factor 23, central pulse pressure, left ventricular ejection fraction, left ventricular end-diastolic diameter, and left ventricular remodeling index compared with the control group (*P* < .05), indicating a more pronounced effect of dapagliflozin on cardiac function. The incidence of adverse drug reactions was low and not significantly different between groups (2.0% in the control group vs 4.0% in the treatment group, *P* > .05). Both dapagliflozin and linagliptin effectively improve glycemic control and cardiac function in patients with T2DM and HF, with favorable safety profiles. However, dapagliflozin appears to confer additional benefits in improving cardiac function, potentially due to its unique pharmacological mechanism.

## 1. Introduction

Type 2 diabetes mellitus (T2DM) is a global public health concern characterized by insulin resistance and impaired insulin secretion.^[1–3]^ Its prevalence continues to increase worldwide, posing a significant risk factor for numerous chronic conditions. As the disease progresses, patients with T2DM often develop multiple complications, including cardiovascular disease, nephropathy, neuropathy, and retinopathy. Among these, heart failure (HF) represents a major and debilitating complication that severely compromises both quality of life and long-term prognosis.^[4,5]^ The incidence of HF is markedly higher in individuals with T2DM, further complicating disease management and treatment strategies.^[6]^ Epidemiological studies have shown that the risk of developing HF in diabetic patients is 2 to 4 times greater than in nondiabetic individuals.^[7,8]^ This elevated risk is attributed not only to the direct cardiotoxic effects of chronic hyperglycemia but also to associated pathophysiological factors such as insulin resistance, hypertension, dyslipidemia, and systemic inflammation. HF significantly increases hospitalization rates and healthcare costs for patients with T2DM while also contributing to reduced survival and diminished quality of life.^[9,10]^ Therefore, identifying and evaluating effective therapeutic approaches for patients with concurrent T2DM and HF is of critical clinical importance.

In recent years, advances in the understanding of the pathophysiology of diabetes and its complications have led to the development of novel antidiabetic agents. Among these, sodium-glucose cotransporter 2 inhibitors and dipeptidyl peptidase-4 inhibitors have shown promising efficacy and safety profiles in the management of T2DM.^[11–14]^ Dapagliflozin, a representative SGLT2 inhibitor, lowers blood glucose by inhibiting SGLT2 in the proximal renal tubules, thereby reducing glucose reabsorption and increasing urinary glucose excretion. Beyond its glycemic benefits, dapagliflozin has demonstrated cardiovascular and renal protective effects.^[15]^ These benefits are thought to be mediated through multiple mechanisms, including blood pressure reduction, weight loss, improved insulin sensitivity, and decreased cardiac and renal workload. Clinical trials have consistently shown that dapagliflozin significantly reduces the risk of HF hospitalization and cardiovascular mortality in patients with T2DM.^[15]^

Linagliptin, a commonly used dipeptidyl peptidase-4 (DPP-4) inhibitor, exerts its glucose-lowering effect by inhibiting the degradation of glucagon-like peptide-1, thereby enhancing insulin secretion and suppressing glucagon release.^[16–18]^ Linagliptin provides effective glycemic control without substantially increasing the risk of hypoglycemia. Moreover, DPP-4 inhibitors have demonstrated modest cardiovascular benefits, potentially through anti-inflammatory effects and improvements in endothelial function.^[19]^

To explore the comparative effects of dapagliflozin and linagliptin on glycemic control and cardiac function in patients with T2DM complicated by HF, we conducted a retrospective analysis of clinical data. By examining changes in glycemic markers and cardiac function indicators, including N-terminal pro–B-type natriuretic peptide (NT-proBNP), fibroblast growth factor 23 (FGF23), and copeptin (CPP), as well as echocardiographic parameters, this study aims to provide real-world evidence to guide clinical decision-making. The incidence of adverse drug reactions was also assessed to evaluate the safety and tolerability of both medications in routine clinical practice.

## 2. Materials and methods

### 2.1. Study design

This study was approved by the Ethics Committee of the Sixth Hospital of Wuhan, Affiliated Hospital of Jianghan University (Approval No. SH20210125). A retrospective study design was employed, involving the division of 200 patients into a control group and an experimental group based on their prior treatment regimens. To ensure comparability and balance of baseline characteristics between the 2 groups, propensity score matching was utilized. Through this method, 100 patients from the experimental group were matched with 100 patients from the control group, thereby minimizing potential confounding variables and enhancing the reliability of the comparative analysis.

Among all primary and secondary outcome measures, glycated hemoglobin (HbA1c) required the largest sample size to detect a statistically significant difference, due to its relatively smaller effect size and larger variability. Therefore, sample size estimation was based on HbA1c to ensure adequate power for the entire study. The sample size was estimated using a 2-sided α of 0.05 and a statistical power of 80%, based on a 2-sample *t* test. Assuming a minimum clinically significant difference of 0.5 units (in HbA1c) and a standard deviation of 1.2, the required sample size was calculated to be ~90 participants per group. Considering a potential 10% data loss due to incomplete records, the final sample size was set at 100 patients per group, totaling 200 participants, to ensure sufficient statistical power and data completeness.


$$\begin{array}{l} n=\frac{{\left(Z_{1-\alpha /2\;}+Z_{1-\beta }\right)}^{2}\times 2\sigma^{2}}{{\left(\mu_{1}-\mu_{2}\right)}^{2}} \end{array}$$

$$\begin{array}{l} \begin{array}{lll} & n=\frac{{\left(1.96+0.84\right)}^{2}\cdot 2\cdot {\left(1.2\right)}^{2}}{{\left(0.5\right)}^{2}}=\frac{{\left(2.8\right)}^{2}\cdot 2\cdot 1.44}{0.25}\; & =\frac{7.84\cdot 2\cdot 1.44}{0.25}=\frac{22.5792}{0.25}=90.3168.\; \end{array} \end{array}$$

### 2.2. Patient selection

The study population comprised patients diagnosed with both T2DM and HF who were admitted to our hospital between January 2021 and December 2023. The inclusion and exclusion criteria were defined as follows:

Inclusion Criteria: (1) Age ≥ 18 years; (2) Previously diagnosed with T2DM, with baseline antidiabetic therapy consisting of metformin monotherapy only; (3) Recently diagnosed with HF (first-time diagnosis), meeting New York Heart Association (NYHA) functional classification II to IV; (3) No prior use of SGLT2 inhibitors, DPP-4 inhibitors, or any guideline-directed HF medications (e.g., β-blockers, angiotensin-converting enzyme inhibitors/angiotensin receptor blockers, aldosterone antagonists) before admission; (4) NYHA functional classification II to IV; and (5) No HF exacerbation in the past 6 months.

Exclusion Criteria: (1) Presence of other cardiac abnormalities, such as valvular heart disease, pericardial disease, etc; (2) Acute diabetic complications, such as diabetic ketoacidosis, lactic acidosis, etc; (3) Concurrent cerebrovascular disease; (4) Concurrent malignant tumors, immune system diseases, or other organ dysfunction; (5) History of allergy to the study-related drugs; and (6) Long-term use of steroids or immunosuppressive drugs.

### 2.3. Drugs, reagents, and instruments

Metformin Tablets: Manufactured by Sino-American Shanghai Squibb Pharmaceuticals Ltd., 0.5 g per tablet, Approval No.: National Medicine Standard H20023370.

linagliptin Tablets: Manufactured by Boehringer Ingelheim (Shanghai) Pharmaceuticals Ltd., 5 mg per tablet, Approval No.: National Medicine Standard HJ20171616.

dapagliflozin Tablets: Manufactured by Boehringer Ingelheim (Shanghai) Pharmaceuticals Ltd., 10 mg per tablet, Approval No.: National Medicine Standard HJ20201008.

NT-proBNP Assay Kit: Provided by Beijing Jian’an Biotechnology Co., Ltd.

FGF23 and CPP enzyme-linked immunosorbent assay (ELISA) Kits: Provided by Shanghai Ruifan Biotechnology Co., Ltd.

AU480 Automated Biochemistry Analyzer: Product of Beckman Coulter, USA.

EPIQ CVx Fully Digital Cardiac Color Ultrasound Diagnostic System: Product of Royal Philips, the Netherlands.

### 2.4. Treatment methods

HF management was individualized based on the recommendations of the Chinese Guidelines for the Diagnosis and Treatment of Heart Failure (2018). Treatment options included β-blockers, angiotensin-converting enzyme inhibitors or angiotensin receptor blockers, aldosterone antagonists, and other medications aimed at improving cardiac function and preventing further progression of HF.

For baseline glycemic control, all patients received metformin at a dose of 0.5 grams twice daily. In addition to metformin, the control group was administered linagliptin at a dose of 5 mg orally once daily, taken before breakfast. The experimental group received dapagliflozin at a dose of 10 mg orally once daily. Both treatment regimens were maintained continuously for 6 months, after which clinical efficacy was evaluated.

### 2.5. Observation indicators and efficacy evaluation

#### 2.5.1. Efficacy criteria

Clinical efficacy was evaluated according to the Guiding Principles for Clinical Research of New Chinese Medicines.^[20]^

#### 2.5.2. Glycemic indicators and cardiac biomarkers

##### 2.5.2.1. Sampling procedure

For all participants, fasting venous blood samples were collected at 2 time points: prior to treatment initiation and after 6 months of therapy. At each time point, 2 separate 3-mL blood samples were obtained to accommodate the distinct processing requirements for glucose and cardiac biomarker assays.

Samples for glucose-related measurements were collected in fluoride anticoagulant tubes to promptly inhibit glycolysis and preserve glucose levels. In contrast, samples for cardiac biomarkers were processed to obtain either serum or appropriately treated plasma, in accordance with the specific requirements of ELISA or chemiluminescence immunoassay techniques. All samples were handled following standardized, assay-specific protocols to ensure the accuracy of measurements and the reliability of the resulting data.

##### 2.5.2.2. Glycemic indicators

Fasting blood glucose (FBG) and 2-hour postprandial blood glucose (2hPBG) levels were determined using the glucose oxidase method. HbA1c was measured using high-performance liquid chromatography.^[21]^

##### 2.5.2.3. Cardiac biomarkers

NT-proBNP was measured by direct chemiluminescence assay. FGF23 and CPP were assessed using ELISA kits.^[22]^

#### 2.5.3. Cardiac function evaluation

Cardiac function was assessed before and after treatment using color Doppler echocardiography.^[23,24]^ Measurements included left ventricular end-diastolic diameter (LVEDD), left ventricular ejection fraction (LVEF), and left ventricular remodeling index (LVRI), obtained from apical 2-chamber and 4-chamber views.

### 2.6. Safety evaluation

Drug safety was monitored throughout the 6-month treatment period. Adverse events (AEs) were assessed and documented at each follow-up visit through a combination of patient self-reports, physician evaluations, and laboratory test reviews. The monitored AEs included, but were not limited to: hypoglycemia, nausea or vomiting, dizziness or hypotension, signs of hepatic or renal impairment, hypersensitivity reactions, and diabetic ketoacidosis.

Events were classified according to severity (mild, moderate, severe) and assessed for potential association with the study drugs. Serious AEs were defined according to International Conference on Harmonisation criteria. All AEs were reviewed by 2 independent clinicians to ensure objectivity.^[25,26]^

### 2.7. Statistical analysis

Statistical analyses were conducted using SPSS version 30.0 (IBM Corp., Armonk). Continuous variables with a normal distribution were expressed as mean ± standard deviation ($\bar{x}\pm s$). Between-group comparisons were performed using independent samples *t* tests, while within-group comparisons (pre- and posttreatment) were analyzed using paired samples *t* tests. Categorical variables were presented as frequencies and percentages (%), and intergroup comparisons were evaluated using the chi-square test.

To assess treatment effects over time and between groups, repeated measures analysis of variance was employed for continuous variables. This method enabled the evaluation of the main effects of time and treatment group, as well as their interaction (time × group), indicating whether the trajectory of change differed between treatment arms. A 2-sided *P*-value < .05 was considered statistically significant.

In addition to *P*-values, effect sizes were calculated to evaluate the practical significance of differences. For categorical data analyzed using chi-square tests, Cohen *w* was applied, with thresholds of 0.1 (small), 0.3 (medium), and 0.5 (large) used to interpret the magnitude of group differences. Effect sizes were reported alongside *P*-values where appropriate to provide a more comprehensive interpretation of the findings.

## 3. Results

### 3.1. General information

A total of 240 patients were initially screened for eligibility. After excluding 40 cases that did not meet the inclusion criteria, 200 patients were ultimately enrolled in the study, with 100 assigned to the control group and 100 to the experimental group. Baseline characteristics, including gender, age, blood glucose levels, blood pressure, and HF classification, showed no statistically significant differences between the 2 groups (all *P* > .05), indicating good comparability. Detailed baseline data are presented in Table 1.

**Table 1:** Comparison of general data in 2 groups.

Variable	Control group (n = 100)	Treatment group (n = 100)	Test statistic	*P*-value
Gender (male/female)	60/40	55/45	χ² = 0.89	.34
Age (years, mean ± SD)	66.5 ± 6.1	63.9 ± 6.3	t = 0.21	.71
BMI (kg/m², mean ± SD)	23.2 ± 2.5	23.3 ± 2.6	t = 0.28	.78
FBG (mg/dL)	170.6 ± 38.3	172.6 ± 41.0	t = 0.34	.73
HbA1c (%)	8.58 ± 1.79	8.66 ± 1.87	t = 0.41	.68
TC (mg/dL)	174.0 ± 27.1	170.1 ± 30.9	t = 1.03	.31
LDL-C (mg/dL)	100.5 ± 19.3	104.4 ± 23.2	t = 1.52	.13
SBP (mm Hg, mean ± SD)	133.3 ± 10.4	134.2 ± 10.1	t = 0.78	.43
DBP (mm Hg, mean ± SD)	82.3 ± 8.0	82.6 ± 7.8	t = 0.29	.77
Smoking history (n,%)	60 (60.00)	55 (55.00)	χ² = 0.78	.38
Cardiac function grade (Ⅱ/Ⅲ/Ⅳ)	30/35/35	25/37/38	χ² = 1.82	.40
Diuretic	70 (70.00)	65 (65.00)	χ² = 0.65	.42
Aldosterone receptor antagonist	90 (90.00)	92 (92.00)	χ² = 0.28	.59
ACEI/ARB	90 (90.00)	88 (88.00)	χ² = 0.17	.68
β-Blocker	85 (85.00)	82 (82.00)	χ² = 0.34	.56

ACEI = angiotensin-converting enzyme inhibitor, ARB = angiotensin receptor blocker, BMI = body mass index, DBP = diastolic blood pressure, FBG = fasting blood glucose, HbA1c = glycated haemoglobin, LDL-C = low-density lipoprotein cholesterol, SBP = systolic blood pressure, TC = total cholesterol.

### 3.2. Comparison of clinical efficacy between 2 groups

After treatment, the total effective rates in the treatment group and control group were 97.50% and 80.00%, respectively. The difference between the 2 groups was statistically significant (*P* < .001), as shown in Table 2. To further assess the practical significance of this difference, we calculated the effect size using Cohen *w*, which was found to be 0.332, indicating a medium effect size according to conventional benchmarks. This suggests that while the statistical difference is significant, the magnitude of the effect should be interpreted with caution, especially in light of the study’s retrospective design and moderate sample size. Therefore, the findings should be viewed as indicating a promising trend, rather than conclusive evidence of superiority, pending further validation in prospective studies.

**Table 2:** Comparison of clinical efficacy between the 2 groups.

Item	Control group (n = 100)	Treatment group (n = 100)	Test statistic	*P*-value	*w*
Markedly effective	30	75	*χ²* = 22.09	<.001*	0.332
Effective	50	65			
Ineffective	20	5			
Total effective rate	80 (80.00%)	140 (97.50%)			

### 3.3. Comparison of blood glucose indexes between 2 groups

Following treatment, both groups exhibited significant reductions in FBG, 2hPBG, and HbA1c compared with their respective baseline levels (all *P* < .05). However, no statistically significant differences were observed between the 2 groups in terms of FBG, 2hPBG, or HbA1c after treatment (all *P* > .05). These findings are summarized in Table 3.

**Table 3:** Comparative changes in glycemic and lipid parameters before and after treatment between groups.

Parameter	Control (before)	Control (after)	Treatment (before)	Treatment (after)	*P* (Time)	*P* (Group)	*P* (Interaction)
FBG (mg/dL)	170.6 ± 38.3	140.2 ± 21.8	172.6 ± 41.0	136.1 ± 20.7	0	.001	.225
2h PBG (mg/dL)	237.8 ± 44.6	161.3 ± 23.8	239.2 ± 46.1	159.7 ± 25.4	0	.985	.553
HbA1c (%)	8.58 ± 1.79	7.59 ± 1.38	8.66 ± 1.87	7.49 ± 1.22	0	.61	.469
TC (mg/dL)	174.0 ± 27.1	166.3 ± 23.2	170.1 ± 30.9	154.7 ± 19.3	.002	.01	.015
LDL-C (mg/dL)	100.5 ± 19.3	96.7 ± 15.5	104.4 ± 23.2	88.9 ± 15.5	.008	.02	.01
HDL-C (mg/dL)	46.4 ± 11.6	50.3 ± 11.6	42.5 ± 11.6	54.1 ± 7.7	.001	.06	.045
TG (mg/dL)	168.3 ± 35.4	150.6 ± 26.6	177.1 ± 44.3	132.9 ± 26.6	0	.015	.007

2h PBG = 2-Hour Postprandial Blood Glucose, FBG = fasting blood glucose, HbA1c = glycated hemoglobin, HDL-C = high-density lipoprotein cholesterol, LDL-C = low-density lipoprotein cholesterol, TC = total cholesterol, TG = triglycerides.

### 3.4. Comparison of cardiac function indexes between 2 groups

After treatment, both groups demonstrated significant improvements in cardiac function. Serum levels of NT-proBNP, FGF23, and CPP significantly decreased from baseline in both the control and treatment groups (all *P* < .05). Echocardiographic parameters, including LVEDD and LVRI, also showed significant reductions, while LVEF increased markedly (*P* < .05).

In addition, newly assessed functional parameters showed consistent improvements: cardiac output (CO) and cardiac index were both significantly elevated after treatment in both groups (*P* < .05), with the treatment group exhibiting significantly greater improvements than the control group (*P* < .05). Furthermore, NYHA functional classification improved significantly in both groups, with a higher proportion of patients in the treatment group improving to class II. Overall, improvements across all cardiac function indicators were significantly more pronounced in the treatment group compared with the control group (all *P* < .05), as detailed in Table 4.

**Table 4:** Comparison of cardiac function indicators.

Parameter	Control (before)	Control (after)	Treatment (before)	Treatment (after)	*P* (time)	*P* (group)	*P* (interaction)
Cardiac function grade (II/III/IV)	30/35/35	42/40/18	25/37/38	58/35/7	.002	.015	.008
NT-proBNP (pg/mL)	2300.56 ± 815.32	1030.45 ± 305.67	2390.78 ± 876.54	600.28 ± 210.34	<.001	<.001	<.001
FGF23 (μg/L)	655.32 ± 84.65	485.34 ± 73.56	675.43 ± 97.85	355.67 ± 61.54	<.001	<.001	<.001
CPP (pmol/L)	24.35 ± 7.08	16.54 ± 4.28	24.89 ± 6.85	12.45 ± 3.36	<.001	<.001	.006
LVEDD (mm)	55.02 ± 2.68	53.15 ± 2.19	55.43 ± 2.76	51.05 ± 2.28	<.001	<.001	<.001
LVEF (%)	47.54 ± 4.05	50.12 ± 3.68	47.12 ± 3.82	52.56 ± 3.29	<.001	<.001	<.001
LVRI (kg/L)	3.25 ± 0.40	2.93 ± 0.31	3.28 ± 0.45	2.60 ± 0.27	<.001	<.001	<.001
CO (L/min)	3.60 ± 0.70	4.10 ± 0.60	3.50 ± 0.80	4.60 ± 0.50	<.001	<.001	<.001
CI (L/min/m²)	2.10 ± 0.30	2.50 ± 0.30	2.00 ± 0.40	2.90 ± 0.30	<.001	<.001	<.001

CI = cardiac index, CO = cardiac output, CPP = copeptin, FGF23 = fibroblast growth factor 23, LVEDD = left ventricular end-diastolic diameter, LVEF = left ventricular ejection fraction, LVRI = left ventricular remodeling index, NT-proBNP = N-terminal pro-brain natriuretic peptide; compared with before treatment.

### 3.5. Safety evaluation

No serious AEs occurred in either group during the treatment period. In the control group, 2 mild AEs were reported: 1 case of headache and 1 case of hypoglycemia (incidence 2.00%). In the treatment group, 4 mild AEs were observed: 1 case each of nausea, vomiting, and hypotension, and 2 cases of hypoglycemia (incidence 4.00%).

All AEs were transient and resolved with routine care. There were no withdrawals due to drug-related events. The overall safety profiles of both dapagliflozin and linagliptin were favorable and comparable, with no statistically significant difference in the incidence of AEs between the 2 groups (*P* > .05).

## 4. Discussion

This study aimed to evaluate the efficacy and safety of dapagliflozin and linagliptin in the treatment of patients with T2DM complicated by HF. The findings demonstrated that both agents effectively reduced blood glucose levels, improved cardiac function parameters, and exhibited comparable safety profiles. In the following discussion, we will elaborate on the key outcomes, interpret their clinical implications, address the study’s limitations, and propose directions for future research.

### 4.1. Analysis of hypoglycemic effect

T2DM is closely linked to cardiovascular complications, particularly HF, due to shared pathophysiological pathways such as chronic hyperglycemia, insulin resistance, systemic inflammation, and endothelial dysfunction.^[27,28]^ Prolonged hyperglycemia contributes not only to microvascular damage but also to myocardial fibrosis, oxidative stress, and neurohormonal activation, thereby accelerating the progression of HF.^[5,6]^

In this context, both linagliptin and dapagliflozin provide glucose-lowering benefits, albeit through distinct mechanisms, with varying implications for cardiovascular health. Linagliptin, a DPP-4 inhibitor, functions by preventing the degradation of glucagon-like peptide-1, thereby enhancing glucose-dependent insulin secretion and inhibiting glucagon release.^[29,30]^ This mechanism improves glycemic control without significantly increasing the risk of hypoglycemia and may offer modest anti-inflammatory and endothelial-protective effects that are relevant in the management of HF.^[19]^

Dapagliflozin, a sodium-glucose cotransporter 2 (SGLT2) inhibitor, reduces blood glucose levels by promoting urinary glucose excretion through inhibition of glucose reabsorption in the proximal renal tubules.^[31]^ In addition to its antihyperglycemic action, dapagliflozin confers multiple cardiometabolic benefits, including reductions in body weight, blood pressure, and plasma volume, as well as improved myocardial energy efficiency. These effects are particularly advantageous in patients with T2DM and HF. In the present study, these benefits were reflected by significantly greater improvements in cardiac biomarkers (e.g., NT-proBNP), left ventricular ejection fraction (LVEF), and CO in the dapagliflozin group compared with the linagliptin group. While both agents were effective in achieving glycemic control, the additional hemodynamic and myocardial benefits observed with dapagliflozin suggest that it may be the more favorable option for patients with T2DM complicated by HF.

### 4.2. Improvement in cardiac function

The pathophysiological mechanisms underlying HF are complex, involving processes such as cardiomyocyte apoptosis, myocardial fibrosis, and structural cardiac remodeling. Patients with HF typically exhibit impaired CO, resulting in inadequate perfusion of vital organs and tissues, which substantially compromises both quality of life and life expectancy. Biomarkers such as NT-proBNP and CPP are widely recognized indicators of HF severity and therapeutic response.^[31,32]^ NT-proBNP is a peptide hormone released in response to increased cardiac wall stress and is strongly associated with HF severity. CPP, a surrogate marker for vasopressin, is linked to myocardial fibrosis and adverse remodeling, and thus serves as an important indicator of changes in cardiac structure and function.^[33]^

In the present study, patients in the dapagliflozin group exhibited greater reductions in NT-proBNP, FGF23, and CPP levels compared with those in the linagliptin group. Additionally, echocardiographic parameters, including reductions in LVEDD and LVRI, along with significant improvements in LVEF, were more pronounced in the dapagliflozin group. These findings suggest a superior effect of dapagliflozin in improving cardiac function.

This result aligns with previous large-scale clinical trials, such as the EMPA-REG OUTCOME study, which demonstrated that SGLT2 inhibitors significantly reduce the risk of cardiovascular death and HF hospitalization. The cardioprotective effects of dapagliflozin are believed to stem from multiple mechanisms, including reduced cardiac preload and afterload, improved myocardial energy metabolism, and attenuation of myocardial fibrosis. These multifactorial benefits may account for the enhanced cardiac outcomes observed in this study.

### 4.3. Safety evaluation

In terms of safety, no severe adverse reactions were observed in either treatment group, and the overall incidence of drug-related AEs was low, indicating that both dapagliflozin and linagliptin were well tolerated in patients with T2DM complicated by HF.^[34–36]^ These findings are consistent with previous studies confirming the favorable safety profile of linagliptin, and they align with recent cardiovascular safety data on SGLT2 inhibitors.

The most commonly reported AEs associated with linagliptin include headache and hypoglycemia, both of which occur at a low incidence and are generally mild in severity. Adverse reactions observed with dapagliflozin included nausea, vomiting, hypotension, and hypoglycemia. Although the incidence of AEs was slightly higher in the dapagliflozin group compared with the linagliptin group, these events remained within an acceptable range and were predominantly mild to moderate in nature.

### 4.4. Study limitations

Although this study has achieved certain results, there are also some limitations. First, the study was conducted at a single center with a relatively small sample size, which may affect the external validity of the results. The limitation of a single-center study is that its results may be limited by specific populations and geographical factors, unable to fully reflect the clinical effect in a wider range. Second, the follow-up period of the study was only 6 months, and the long-term efficacy and safety still need further observation. HF and diabetes are chronic diseases, and short-term studies cannot fully evaluate the long-term impact of drugs. Additionally, this study did not strictly control factors such as diet and exercise in patients, which may have a certain impact on the study results. The role of lifestyle interventions in improving blood sugar and cardiac function has been widely demonstrated, and failure to strictly control these factors may lead to confusion in the study results.

### 4.5. Future research directions

Future studies should aim to expand the sample size and adopt multicenter, randomized controlled trial designs with long-term follow-up to further validate the findings of this study. Additionally, it is important to investigate the effects of different dosages and administration regimens of dapagliflozin and linagliptin in patients with T2DM complicated by HF. Particular attention should be given to the heterogeneity of HF subtypes, as there are notable differences in pathophysiological mechanisms and therapeutic responses between HF with reduced ejection fraction and HF with preserved ejection fraction. Evaluating the differential efficacy of these agents across HF subtypes would provide valuable insights for individualized treatment strategies. Moreover, further in-depth research is warranted to elucidate the underlying mechanisms of action of these drugs, particularly their roles in improving cardiac function and reversing cardiac remodeling at the molecular and cellular levels. Investigating their specific effects on myocardial energy metabolism, anti-inflammatory pathways, and antioxidant mechanisms may offer a theoretical foundation for the development of novel therapeutic strategies and enhance our understanding of their clinical value in managing T2DM with HF.

In summary, both dapagliflozin and linagliptin can effectively lower blood glucose levels and improve cardiac function in patients with T2DM combined with HF, with good safety profiles. Dapagliflozin may have more advantages in improving cardiac function, but more studies are needed to further verify this. Through future large-scale, multicenter, long-term follow-up studies, we hope to further clarify the long-term efficacy and safety of these drugs, providing a more solid basis for clinical treatment.

## Acknowledgments

We would like to thank the participants of this study for sharing their experiences.

## Author contributions

**Conceptualization:** Wenxin Zai, Ting Dai, Bo Liu.

**Data curation:** Wenxin Zai, Ting Dai, Bo Liu.

**Funding acquisition:** Ting Dai.

**Investigation:** Wenxin Zai, Ting Dai, Li Wang, Bo Liu.

**Methodology:** Wenxin Zai, Ting Dai, Bo Liu.

**Supervision:** Li Wang.

**Validation:** Li Wang.

**Writing – original draft:** Wenxin Zai, Bo Liu.

**Writing – review & editing:** Wenxin Zai, Bo Liu.

## References

[1] Goldstein BJ. Insulin resistance as the core defect in type 2 diabetes mellitus. Am J Cardiol. 2002;90:3–10. 10.1016/s0002-9149(02)02553-5 12231073

[2] Galicia-Garcia U Benito-Vicente A Jebari S. Pathophysiology of type 2 diabetes mellitus. Int J Mol Sci. 2020;21:6275. 32872570 10.3390/ijms21176275 PMC7503727

[3] DeFronzo RA Ferrannini E Groop L. Type 2 diabetes mellitus. Nat Rev Dis Primers. 2015;1:1–22. 10.1038/nrdp.2015.19 27189025

[4] Pop-Busui R Januzzi JL Bruemmer D. Heart failure: an underappreciated complication of diabetes. A consensus report of the American Diabetes Association. Diabetes Care. 2022;45:1670–90. 35796765 10.2337/dci22-0014 PMC9726978

[5] Kenny HC Abel ED. Heart failure in type 2 diabetes mellitus: impact of glucose-lowering agents, heart failure therapies, and novel therapeutic strategies. Circ Res. 2019;124:121–41. 30605420 10.1161/CIRCRESAHA.118.311371 PMC6447311

[6] Low Wang CC Hess CN Hiatt WR Goldfine AB. Clinical update: cardiovascular disease in diabetes mellitus: atherosclerotic cardiovascular disease and heart failure in type 2 diabetes mellitus–mechanisms, management, and clinical considerations. Circulation. 2016;133:2459–502. 27297342 10.1161/CIRCULATIONAHA.116.022194 PMC4910510

[7] Abdissa SG Deressa W Shah AJ. Incidence of heart failure among diabetic patients with ischemic heart disease: a cohort study. BMC Cardiovasc Disord. 2020;20:1–9. 32306907 10.1186/s12872-020-01457-6 PMC7169007

[8] Rosano GM Vitale C Seferovic P. Heart failure in patients with diabetes mellitus. Card Fail Rev. 2017;3:52–5. 28785476 10.15420/cfr.2016:20:2 PMC5494155

[9] Ponikowski P Anker SD AlHabib KF. Heart failure: preventing disease and death worldwide. ESC Heart Fail. 2014;1:4–25. 28834669 10.1002/ehf2.12005

[10] Cavender MA Steg PG Smith SC Jr. Impact of diabetes mellitus on hospitalization for heart failure, cardiovascular events, and death: outcomes at 4 years from the Reduction of Atherothrombosis for Continued Health (REACH) Registry. Circulation. 2015;132:923–31. 26152709 10.1161/CIRCULATIONAHA.114.014796

[11] Deng S Zhao H Chai S. Influence of early use of sodium-glucose transport protein 2 inhibitors, glucagon-like peptide-1 receptor agonists and dipeptidyl peptidase-4 inhibitors on the legacy effect of hyperglycemia. Front Endocrinol. 2024;15:1369908. 10.3389/fendo.2024.1369908 PMC11128627 38803473

[12] Sim AY Barua S Kim JY Lee Y-H Lee JE. Role of DPP-4 and SGLT2 inhibitors connected to Alzheimer disease in type 2 diabetes mellitus. Front Neurosci. 2021;15:708547. 34489627 10.3389/fnins.2021.708547 PMC8417940

[13] Faruqui AA. Combination of sodium-glucose co-transporter 2 inhibitors and dipeptidyl peptidase-4 inhibitor: a complementary approach to diabetes management. Asian J Res Rep Endocrinol. 2022;5:35–46.

[14] Rokszin G Kiss Z Sütő G. Sodium-glucose co-transporter 2 inhibitors may change the development of urinary tract and hematological malignancies as compared with dipeptidyl peptidase-4 inhibitors: data of the post-hoc analysis of a nationwide study. Front Oncol. 2021;11:725465. 34778040 10.3389/fonc.2021.725465 PMC8581296

[15] Ni L Yuan C Chen G Zhang C Wu X. SGLT2i: beyond the glucose-lowering effect. Cardiovasc Diabetol. 2020;19:98. 32590982 10.1186/s12933-020-01071-y PMC7320582

[16] Grunberger G. Clinical utility of dipeptidyl peptidase-4 inhibitors: a descriptive summary of current efficacy trials. Eur J Clin Pharmacol. 2014;70:1277–89. 25176337 10.1007/s00228-014-1727-5

[17] Lietzau G Magni G Kehr J. Dipeptidyl peptidase-4 inhibitors and sulfonylureas prevent the progressive impairment of the nigrostriatal dopaminergic system induced by diabetes during aging. Neurobiol Aging. 2020;89:12–23. 32143981 10.1016/j.neurobiolaging.2020.01.004

[18] Yang J Huang C Wu S. The effects of dipeptidyl peptidase-4 inhibitors on bone fracture among patients with type 2 diabetes mellitus: a network meta-analysis of randomized controlled trials. PLoS One. 2017;12:e0187537. 29206832 10.1371/journal.pone.0187537 PMC5716604

[19] Aroor AR Sowers JR Jia G DeMarco VG. Pleiotropic effects of the dipeptidylpeptidase-4 inhibitors on the cardiovascular system. Am J Physiol Heart Circ Physiol. 2014;307:H477–92. 24929856 10.1152/ajpheart.00209.2014 PMC4137125

[20] Xiaoyu Z. Guiding principles for clinical research of new Chinese medicine. China Medical Science and Technology Publishing; 2002.

[21] Grady M Katz LB Levy BL. Use of blood glucose meters featuring color range indicators improves glycemic control in patients with diabetes in comparison to blood glucose meters without color (ACCENTS Study). J Diabetes Sci Technol. 2018;12:1211–9. 29848106 10.1177/1932296818775755 PMC6232745

[22] Bojic M Koller L Cejka D Niessner A Bielesz B. Propensity for calcification in serum associates with 2-year cardiovascular mortality in ischemic heart failure with reduced ejection fraction. Front Med (Lausanne). 2021;8:672348. 34222283 10.3389/fmed.2021.672348 PMC8249741

[23] Chen YZ Qiao SB Hu FH. Left ventricular remodeling and fibrosis: sex differences and relationship with diastolic function in hypertrophic cardiomyopathy. Eur J Radiol. 2015;84:1487–92. 26001434 10.1016/j.ejrad.2015.04.026

[24] Kan A Fang Q Li S. The potential predictive value of cardiac mechanics for left ventricular reverse remodelling in dilated cardiomyopathy. ESC Heart Fail. 2023;10:3340–51. 37697922 10.1002/ehf2.14529 PMC10682859

[25] Myvizhi Selvi S. Prospective Study in Diabetic Patients to Monitor Adverse Drug Reactions in Various Class of Anti-Diabetic Drugs. Padmavathi College of Pharmacy and Research Institute; 2014.

[26] Hossain MA Pervin R. Current antidiabetic drugs: review of their efficacy and safety. In: Nutritional and Therapeutic Interventions for Diabetes and Metabolic Syndrome;2018:455–73.

[27] Dal Canto E Ceriello A Rydén L. Diabetes as a cardiovascular risk factor: an overview of global trends of macro and micro vascular complications. Eur J Prev Cardiol. 2019;26(2_suppl):25–32. 31722562 10.1177/2047487319878371

[28] Zakir M Ahuja N Surksha MA. Cardiovascular complications of diabetes: from microvascular to macrovascular pathways. Cureus. 2023;15:e45835. 37881393 10.7759/cureus.45835 PMC10594042

[29] Shah P Ardestani A Dharmadhikari G. The DPP-4 inhibitor linagliptin restores β-cell function and survival in human isolated islets through GLP-1 stabilization. J Clin Endocrinol Metab. 2013;98:E1163–72. 23633194 10.1210/jc.2013-1029

[30] Gallwitz B. Clinical use of DPP-4 inhibitors. Front Endocrinol. 2019;10:464622. 10.3389/fendo.2019.00389 PMC6593043 31275246

[31] Whaley JM Tirmenstein M Reilly TP. Targeting the kidney and glucose excretion with dapagliflozin: preclinical and clinical evidence for SGLT2 inhibition as a new option for treatment of type 2 diabetes mellitus. Diabetes Metab Syndr Obes. 2012;5:135–48. 22923998 10.2147/DMSO.S22503 PMC3422910

[32] Alaaraji SFT Awad MM Ismail MA. Study the association of asprosin and dickkopf-3 with KIM-1, NTpro-BNP, GDF-15 and CPP among male iraqi with chronic kidney disease. Syst Rev Pharm. 2020;11:10–7.

[33] Svedung Wettervik T Howells T Hånell A Nyberg C Ronne-Engström E. NT-proBNP and troponin I in high-grade aneurysmal subarachnoid hemorrhage: relation to clinical course and outcome. J Crit Care. 2022;72:154123. 35908328 10.1016/j.jcrc.2022.154123

[34] Iqbal N Allen E Öhman P. Long-term safety and tolerability of saxagliptin add-on therapy in older patients (aged≥ 65 years) with type 2 diabetes. Clin Interv Aging. 2014;9:1479–87. 25214775 10.2147/CIA.S68193 PMC4158996

[35] Bolen S Feldman L Vassy J. Systematic review: comparative effectiveness and safety of oral medications for type 2 diabetes mellitus. Ann Intern Med. 2007;147:386–99. 17638715 10.7326/0003-4819-147-6-200709180-00178

[36] Leporini C Piro R Ursini F. Monitoring safety and use of old and new treatment options for type 2 diabetic patients: a two-year (2013–2016) analysis. Expert Opin Drug Saf. 2016;15:17–34. 27718744 10.1080/14740338.2016.1246531

